# On my eight articles with the Professor Jorge Sotomayor

**DOI:** 10.1007/s40863-024-00434-7

**Published:** 2024-05-22

**Authors:** Jaume Llibre

**Affiliations:** Departament de Matemàtiques, Universitat Autònoma de Barcelona, 08193 Bellaterra, Barcelona, Catalonia Spain

## Abstract

In this note I summarize the eight articles that I wrote with the Professor Jorge Sotomayor and the consequences of some these articles.

The existence of Professor Sotomayor arrived to me at the earliest 1980s through his excellent book *Lições de equações diferenciais ordinárias* published in 1979. Nowdays I continue using parts of his book when I teach differential equations.

Few years after I got his other good book *Curvas definidas por equações diferenciais no plano* published in 1981. This book helped strongly to our group in Barcelona because it introduced us to work on the polynomial differential equations, one of the main areas of research of our group since then.

Approximately around the middle of the years 1980s Sotomayor visited my university invited by our colleague Professor Carles Perello. This gaves me the opportunity of knowing him personally. Since that moment we always have been in contact. I visited him several times, first in IMPA and later on in USP, and he visited me and our group several times in Barcelona. These last years my contacts with Sotomayor were mainly during the meetings of the *Oficina de Sistemas Dinâmicos*.

Due to my contacts with Sotomayor our group started to collaborate initially with the Professors Carlos Gutierrez, after with Professor Marco Antonio Teixeira, and step by step with all our present big number of collaborators in Brazil. So we have a big mathematical debt and a big friendship debt with Soto, as all his friends called him.

My first two papers with Soto were together with Professor Armengol Gasull:

A. GASULL, J. LLIBRE AND J. SOTOMAYOR, *Limit cycles of vector fields of the form* $X(v)=Av+f(v)Bv$, J. Differential Equations **67** (1987), 90–110.

A. GASULL, J. LLIBRE AND J. SOTOMAYOR, *Further considerations on the number of vector fields of the form* $X(v)=Av+f(v)Bv$, J. Differential Equations **68** (1987), 36–40.

Soto was motivated to wrote these two papers because these class of differential systems was previously studied by Professor Carmen Chicone as an important extension of a less general class of quadratic polynomial differential systems considered by D. E. Koditschek and K. S. Narendra.

In the first paper we characterize under convenient assumptions the limit cycles of those differential systems when at the origin the system has a focus, and in the second one when at the origin the system has a node with different eigenvalues.

These two papers woke me up the interest for studying the limit cycles of the differential systems, one of my favourite topics of research.

My third paper with Soto also was with Armengol Gasull:

A. GASULL, J. LLIBRE AND J. SOTOMAYOR, *Global asymptotic stability for differential equations in the plane*, J. Differential Equations **91** (1991), 327–335.

This was probably my best paper with Soto.

In this paper we consider an autonomous system of differential equations

1 $$\begin{aligned} \dot{x}=\frac{dx}{dt}=f(x), \end{aligned}$$

where $$x=(x_1, x_2)$$ and $$f(x)= (f_1(x_l, x_2),f_2(x_1, x_2))$$.

Let $\mathcal {F}$ be the class of $C^1$ maps $f:\mathbb {R}^2 \rightarrow \mathbb {R}^2$ such that

(i) the origin $O = (0, 0)$ is an equilibrium point of system (1), i.e. $f(O) = O$,
(ii) tr $Df(x) < 0$ on $\mathbb {R}^2$,
(iii) det $Df(x) > 0$ on $\mathbb {R}^2$,

where $Df(x) = (\partial f_i/\partial x_j)$ is the Jacobian matrix of the map *f*.

Now we define some problems.

*Fundamental Problem on Global Asymptotic Stability.* Does $f\in \mathcal {F}$ imply that $x = O$ is a global asymptotically stable solution of the differential system (1)? In other words, does every orbit of system (1) approach *O* as $t \rightarrow \infty$?

## Problem 1

Does $f\in \mathcal {F}$ imply that the mapping $f:\mathbb {R}^2 \rightarrow \mathbb {R}^2$ is globally one-to-one?

## Problem 2

Does $f\in \mathcal {F}$ imply that there is a natural number *K* such that for each $p\in \mathbb {R}^2$ the number of solutions of $f(x) = p$ is bounded by *K*?

## Problem 3

Does $f\in \mathcal {F}$ imply that there are two positive constants $\rho$ and *r* such that $|f(x)| \ge \rho >O$ for $|x|\ge r>O$ (where | | denotes the Euclidean norm)?

## Problem 4

Does $f\in \mathcal {F}$ imply that

$$\begin{aligned} \int _0^\infty [\min _{|x|=r} |f(x)|] dr= \infty ? \end{aligned}$$

The main result of that paper was the following:

## Theorem

The following five statements are equivalent.

(FP): The Fundamental Problem has an affirmative answer for all $f\in \mathcal {F}$.
(Pi): Problem *i* has an affirmative answer for all $f\in \mathcal {F}$, where $i\in \{ 1, 2, 3, 4\}$.

Carlos Gutierrez told me that was this paper with Soto and Armengol that motivates him to write one of his best works:

C. GUTIERREZ, *A solution to the bidimensional global asymptotic stability conjecture*, Ann. Inst. Henri Poincaré **12** (1995), 627–671.

My next paper with Soto was:

J. LLIBRE AND J. SOTOMAYOR, *Phase portraits of planar control systems*, Nonlinear Analysis, Theory, Methods and Applications **27** (1996), 1177–1197.

In this paper we characterize the limit cycles of the planar non-smooth differential systems

$$\begin{aligned} \dot{x} = Ax+\varphi (k\cdot x)b, \end{aligned}$$

where *A* is a $2\times 2$ real matrix, $x,k,b\in \mathbb {R}^2$ and

$$\begin{aligned} \varphi (v)= \left\{ \begin{array}{ll} -u &{}\quad \text{ for }\ v\le -u, \\ v &{}\quad \text{ for }\ -u\le v \le u, \\ u &{} \quad \text{ for }\ v\ge u, \end{array}\right. \end{aligned}$$

with $$u>0$$.

These differential systems are important in the direct control theory.

This paper was my first paper on non-smooth differential systems.

Just after doing this paper I wrote with Professor Enrique Ponce the paper:

J. LLIBRE AND E. PONCE, *Global first harmonic bifurcation diagram for odd piecewise linear control systems*, Dynam. Stability Systems **11**(1996), no. 1, 49–88.

This paper was the first paper that the school of Sevilla wrote on non-smooth differential systems. This paper motivates to the school of Sevilla to work on the non-smooth differential systems and to produce the big number of excellent papers on those systems that they have done until now. So the influence of Soto was great and strong.

Always it was Soto who propose me the work to do with him, with only one exception the paper:

J. CHAVARRIGA, J. LLIBRE AND J. SOTOMAYOR, *Algebraic solutions for polynomial systems with emphasis in the quadratic case*, Expositiones Mathematicae **15** (1997), 161–173.

In this paper we improve the classical Darboux theory of integrability of the polynomial differential system, showing how to use a class of equilibrium points of those systems for simplifying their integrability.

My six paper with Soto was:

J. LLIBRE AND J. SOTOMAYOR, *Structural stability of constrained polynomial systems*, Bulletin of the London Math. Soc. **30** (1998), 589–595.

In this paper we characterize the structural stability of the constrained polynomial differential systems of the form

$$\begin{aligned}{} & {} a(x,y)\dot{x}+b(x,y)\dot{y}=f(x,y),\\{} & {} c(x,y)\dot{x}+d(x,y)\dot{y}=g(x,y), \end{aligned}$$

under small perturbations of the coefficients of the polynomial functions *a*, *b*, *c*, *d*, *f* and *g*. These systems differ from the ordinary differential equations at the “impasse points” defined by $ad-bc= 0$. We extended to these differential systems the results on structural stability of the smooth constrained differential systems and of the ordinary polynomial differential systems.

My penultimate work with Soto was also with Professor Michail Zhitomirskii:

J. LLIBRE, J. SOTOMAYOR AND M. ZHITOMIRSKII, *Impasse bifurcations of constrained systems*, in Differential Equations and Dynamical Systems, Fields Institute Communications, Amer. Mat. Soc. **31** (2002), 235–255.

In this paper we studied the generic bifurcations of the families of constrained $C^r$ differential systems of the form

$$\begin{aligned}{} & {} a(x,y,\lambda )\dot{x}+b(x,y,\lambda )\dot{y}=f(x,y,\lambda ),\\{} & {} c(x,y,\lambda )\dot{x}+d(x,y,\lambda )\dot{y}=g(x,y,\lambda ), \end{aligned}$$

where $(x,y)\in \mathbb {R}^2$ and $\lambda$ is a real parameter. The analysis of the bifurcations is located around the impase surface $ad-bc=0$ where the constrained systems differ from the ordinary differential systems.

Finally, my last paper with Soto was also written with Professor Ronaldo Garcia:

A. GASULL, J. LLIBRE AND J. SOTOMAYOR, *Lines of principal curvature on canal surfaces in* $\mathbb {R}^3$, An. Acad. Brasil. Ciênc. 78 (2006), no. 3, 405–415.

In this paper we determined the principal curvatures and principal curvature lines on canal surfaces which are the envelopes of families of spheres with variable radius and centers moving along a closed regular curve in $\mathbb {R}^3$. By means of a connection of the differential equations for these curvature lines and real Riccati equations, it is established that canal surfaces have at most two isolated periodic principal lines. Examples of canal surfaces with two simple and one double periodic principal lines were given.

Of course the lines of curvatures was one of the main topics that Soto dedicated his research with the collaboration of Ronando Garcia and Carlos Gutierrez.

